# Systemic therapy of MSCs in bone regeneration: a systematic review and meta-analysis

**DOI:** 10.1186/s13287-021-02456-w

**Published:** 2021-07-02

**Authors:** Jingfei Fu, Yanxue Wang, Yiyang Jiang, Juan Du, Junji Xu, Yi Liu

**Affiliations:** Laboratory of Tissue Regeneration and Immunology and Department of Periodontics, Beijing Key Laboratory of Tooth Regeneration and Function Reconstruction, School of Stomatology, Capital Medical University, Tian Tan Xi Li No.4, Beijing, 100050 People’s Republic of China

**Keywords:** Mesenchymal stem cells, Bone regeneration, Systemic treatment, Meta-analysis

## Abstract

**Objectives:**

Over the past decades, many studies focused on mesenchymal stem cells (MSCs) therapy for bone regeneration. Due to the efficiency of topical application has been widely dicussed and systemic application was also a feasible way for new bone formation, the aim of this study was to systematically review systemic therapy of MSCs for bone regeneration in pre-clinical studies.

**Methods:**

The article search was conducted in PubMed and Embase databases. Original research articles that assessed potential effect of systemic application of MSCs for bone regeneration in vivo were selected and evaluated in this review, according to eligibility criteria. The efficacy of MSC systemic treatment was analyzed by random effects meta-analysis, and the outcomes were expressed in standard mean difference (SMD) and its 95% confidence interval. Subgroup analyses were conducted on animal species and gender, MSCs types, frequency and time of injection, and bone diseases.

**Results:**

Twenty-three articles were selected in this review, of which 21 were included in meta-analysis. The results showed that systemic therapy increased bone mineral density (SMD 3.02 [1.84, 4.20]), bone volume to tissue volume ratio (2.10 [1.16, 3.03]), and the percentage of new bone area (7.03 [2.10, 11.96]). Bone loss caused by systemic disease tended to produce a better response to systemic treatment (p=0.05 in BMD, p=0.03 in BV/TV).

**Conclusion:**

This study concluded that systemic therapy of MSCs promotes bone regeneration in preclinical experiments. These results provided important information for the systemic application of MSCs as a potential application of bone formation in further animal experiments.

**Supplementary Information:**

The online version contains supplementary material available at 10.1186/s13287-021-02456-w.

## Introduction

The problem of bone regeneration has always been a hot topic of research. In many cases, such as bone defect, fracture, osteoporosis, and osteonecrosis, bone regeneration is an urgent problem to be solved [1–4]. As we all know, bone undergoes continuous remodeling during life. Healthy bone remodeling includes both bone formation by osteoblasts and resorption by osteoclasts. An intricate balance between the activities of osteoblasts and osteoclasts determines the health of bone [5, 6]. Normally, small bone defects can be effectively repaired. However, in some cases, this balance may be disrupted, our body cannot maintain self-regenerate, and clinical treatment is needed [7, 8]. Therefore, further intervention in bone tissue engineering is required. For orthopedic and craniofacial surgeons, achieving complete and functional bone regeneration remains a major challenge.

A variety of techniques are used in the clinic for bone regeneration, such as bone grafting, distraction osteogenesis, and guided bone regeneration [9–11]. Several treatment methods have achieved clear therapeutic effects, especially autogenous bone grafts. Autogenous bone grafts is the gold standard for bone regeneration due to the osteoinductive, bone conductivity, and histocompatibility of autologous bone [12]. However, there are still several shortcomings of the gold standard such as extended recovery time, unpredictable absorption, and dependence of the donor’s bone quality and available bone size [13]. As the improvement of comprehension of bone tissue biology as well as current advances in the development of tissue engineering, mesenchymal stem cell (MSCs) therapy has become a hot topic in enhancing bone tissue reconstruction [8, 14, 15]. The main source of MSCs is bone marrow. In addition, they can also be isolated and identified from adipose tissue, peripheral blood, placenta, and other tissues [15–17]. MSCs play a vital role in bone formation [8], and bone is formed via endochondral and intramembranous ossification [18]. On the one hand, MSC-driven condensation occurs firstly, and then MSCs differentiate into chondrocytes during the process of formation of growth plates, which are replaced by new bone in longitudinal-endochondral bone growth. This type of healing mostly occurs in large bone defect with less mechanical stability, initiating the recruitment of MSCs from the periosteum, bone marrow, and circulation [19–21]. On the other hand, MSCs can also directly differentiate to osteoblasts in bone formation such as skull, facial bones, and pelvis, generating by intramembranous ossification without a cartilaginous template [22, 23]. This type of healing usually occurs in minimal bone defects and fractures within the bone metaphysis [21, 24]. However, in recent years, scholars have reported that in patients with osteoporosis, MSCs tend to differentiate into adipocytes rather than osteoblast, leading to disorders of bone formation [25].

Above all, majority of researches are devoted to application of MSCs in bone regeneration. Most researched treatments are local injection or local application of MSCs combined with scaffold [26–28]. However, local strategies still have some limitations, especially in systemic diseases such as multiple fractures and osteoporosis [29]. In these cases, systemic application is easier and more suitable for these patients. To the best of our knowledge, several systematic reviews have been published on the local application of MSCs for bone regeneration [30–32]. Nevertheless, no one focus on systemic application. This systematic review would help provide sufficient evidence to prove the therapeutic potential of systemic applying MSCs to regenerate animal bone tissues and clarify the limitations of existing studies.

## Materials and methods

### Eligibility criteria

#### Type of studies

All preclinical controlled animal model studies with systemic treatment of MSCs for bone regeneration were eligible for this review. Abstracts, reviews, letters, and PhD theses were excluded.

#### Type of participants

All kinds of animals were selected in this review irrespective of type, sex, and age. And any type of MSCs was considered in this systematic review such as bone marrow-derived mesenchymal stem cells (BMSCs), adipose-derived mesenchymal stem cells (ADSCs), gingiva-derived mesenchymal stem cells (GMSCs), dental pulp stem cells (DPSCs), and so on.

#### Type of intervention

Systemic application was compared with control treatment, including intravenous (IV) injection through tail vein or ear vein, intra-ventricular injection, bone marrow cavity’s injection, and so on. Studies utilizing only local application were excluded.

#### Outcome measures

To verify new bone formation, most researches adopted the measurement of bone volume (BV) to tissue volume (TV) ratio, bone mineral density (BMD), and percentage of new bone area. And the methods of measurement were comprised of histological analysis (such as haematoxylin and eosin) and radiographic evaluation (such as computed tomography [CT] scans and micro-CT).

### Database search protocol

A comprehensive search of literature published up to July 2020 was performed in electronic databases PubMed and Embase. Four components were involved in the search strategy: bone regeneration, MSCs, systematic application, and animals (see Supplementary Table S1 for complete search strategy, Additional File 1).

### Study screening

For selection of literatures, two independent reviewers screened the titles and abstracts of literatures independently according to the following eligibility criteria: bone regeneration, in vivo animal studies, original paper, and systemic therapy. Full texts of eligible publications were obtained for further independent evaluation. Any disagreement was solved by discussion or consultation with a third reviewer. Besides, reviews recorded reasons for each rejecting study. All of the progress of screening was performed in Rayyan, the systematic reviews web app (https://rayyan.qcri.org).

### Data extraction process

Data were extracted independently by two reviewers, with disagreements resolved by discussion with a third reviewer. Data was extracted from the full texts of included literatures on: author(s), year, species, age, sex of animals, number of animals per group, animal model, type of MSCs, cell passage number, number of cells, number of cells/kg, the way of administration, application protocol of MSCs (time, frequency of injection), treatment duration, quantification of new bone, combination treatment, and cellular fate of MSCs.

### Quality assessment and risk of bias

Quality assessment of selected articles was performed independently by two reviewers, and the names of the authors, institutions, and journal titles were blinded. Systematic Review Centre for Laboratory animal Experimentation (SYRCLE) Risk of bias (RoB) tool for animal studies was modified and used to performed RoB assessment [33], which was judged as “high,” “low,” or “unclear.” Two questions were added to overcome the problem of scoring excessive “unclear RoB” due to poor reporting details of the include experiments: “1) was it stated that the experiment was randomized at any level?2) was it stated that the experiment was blinded at any level?” [34, 35]. Any disagreement between the reviewers was resolved by discussion and consensus.

### Data synthesis and statistical analysis

Data were analyzed using Review Manager. The primary outcomes BMD between experimental and control groups was performed by meta-analysis, calculating the standardized mean difference (SMD). Besides, BV/TV and percentage of new bone area were also analyzed in this review. If one reference studied with different treatment duration, we just analyzed the longest follow-up time point. I^2^ statistic assessed statistical heterogeneity among included studies, and a random effects model was used if high heterogeneity existed. Forest plots were selected to graphically display effect sizes and their confidence intervals (CI). Subgroup analysis and investigation of heterogeneity were used to explore possible sources of heterogeneity. And subgroup analysis was carried out based on following factors: animal species and gender, the type of cells, frequency and time of injection, and bone diseases. But when we analyzed the gender subgroup, studies with animal models of ovariectomy (OVX)-induced bone loss were excluded because of the limited gender. In addition, it was performed only if it contains at least four independent experiments.

## Results

### Selection of the studies

**Fig. 1:**
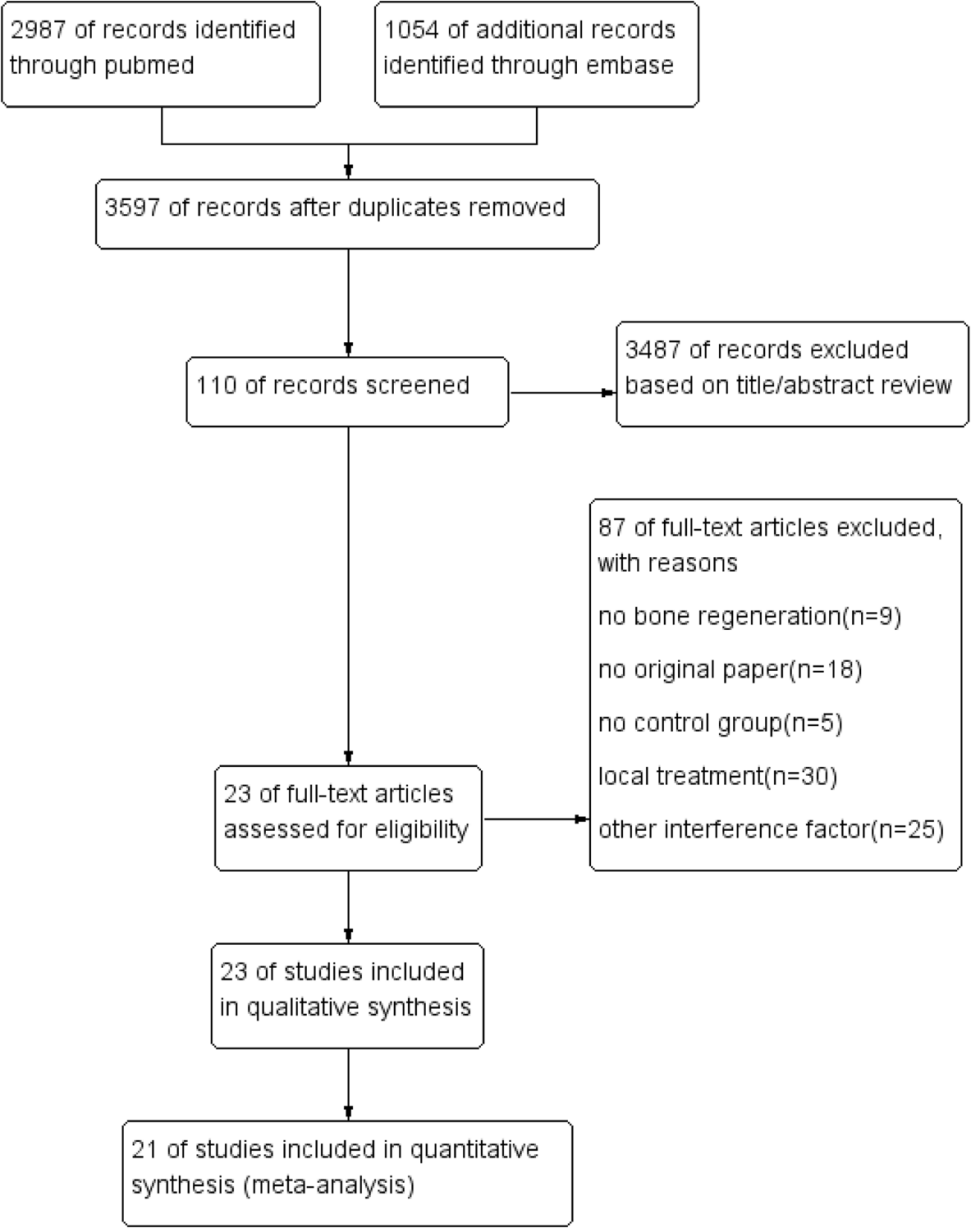
Flowchart for study screening and selection

After duplication in Endnote, the initial search in the PubMed and Embase databases resulted in 3579 papers. After initial screening based on titles and abstracts in Rayyan, 110 publications were obtained for analyzation of full text. Eventually, 23 articles were included after full-text screening [36–58], and 21 studies were selected for meta-analysis (Fig. 1). Two trials [43, 58] were withheld from meta-analysis due to the quantification of new bone was not involved in BV/TV, BMD, or the percentage of newly formed bone.

Study characteristics of selected papers are displayed in Table 1. Four different animal species were used: most commonly used species was mice (15 studies), followed by rat (5 studies), swine (2 studies), and dog (1 study). Male and female animals were approximately equally used in these researches and 2 papers did not mention the gender of animals. Various animal models of bone disease were involved, including different sizes of bone defects (5 studies), different types of bone fractures (9 studies), and different causes of bone loss including osteoporosis, ovariectomy, and osteonecrosis (9 studies). BMSCs were evaluated for bone regeneration in most studies (16 studies), and other five different cell types were also reported, including DSCs (1 study), GMSCs (1 study), ADSCs (2 studies), uterine stem cell-derived osteoprogenitor cells (1 study), umbilical cord blood (UCB)-MSCs (1 study), and MSCs of uncertain origin (1 study). Moreover, both passage number and cell dosing varied greatly across the researches, ranging from passage 1 to passage 8, from 3 × 10^4^ to 4 × 10^9^ cells. Generally, IV injection is the most frequently applied method for systemic therapy (20 studies), and other studies also used bone marrow cavity’s injection (1 study) and intra-ventricular injection (2 studies). Most researches used a single injection (18 studies), and other researches used two (2 studies), four (1 study), five (1 study), or eight (1 study) injections. In the majority of cases, MSCs were injected after surgery (16 studies) or during surgery (5 studies), and only one study used MSC injection before the femoral fracture model was constructed. Moreover, the heterogeneity between the combination treatments was discovered among articles (8 studies), and only LLP2A-Alendronate was mentioned twice.

**Table 1:** Summary of study characteristics in animal model

Author/year	Species	Age	Weight	Sex	N	Animal model	Type of MSCs	Cell passage number	Number of cells	Number of cells/kg	The way of administration	Frequency of injection	Time of injection	Treatment duration	Quantification of new bone	Combination treatment	Cellular fate
Li et al., 2019 [38]	SD rats	12w	250–300g	Female	16	Tibia bone defect	BMSCs	N	3 × 10^4^	1–1.2 × 10^5^/kg	Tail vein	Once	At the same time of transplantation	4w 8w	Percentage of new bone area, BV/TV, BMD	Erythropoietin	Homing to the defect areas
Liu et al., 2014 [37]	Beagle dogs	3–4y	8.5–10kg	Female	4	Mandibular defect	BMSCs	P3	3.4–4 × 10^9^	4 × 10^8^/kg	Bone marrow cavity	Once	At the same time of transplantation	6w	Width of new bone region, mineralized bone area percent	N	Homing to the mandibular defect
Wu et al., 2016 [36]	Balb-c mice	6–8w	N	Male	4	Periapical lesion	hDPSCs	P4	2 × 10^6^	N	Tail vein	Once	After 14 days of pulp exposure	14d	BV/TV	Hypoxic preconditioning	Homing to periapical lesion
Xu et al., 2014 [39]	C57BL/6 mice	7w	N	Male	18	Mandibular defect	GMSCs	P5	1 × 10^6^	N	Tail vein	Once	At the same time of transplantation	2/3w	New bone area	N	Homing to the bone defect
Cheung et al., 2013 [49]	SD rats	3m	N	Female	5	Unilateral closed femoral fractures	MSCs	N	4 × 10^5^	N	Left ventricle injection	Once	After 3 days of fracture	1/2/3/4w	Mean average callus width/area, BV/TV	Low intensity pulsed ultrasound	Homing to the fracture site
Huang et al., 2015 [51]	FVB/N mice	8w	25–35g	Male	10	Open transverse femoral fracture	BMSCs	P4-P8	5 × 10^5^	1.4–2 × 10^7^/kg	Left ventricle injection	Once	After 4 days of fracture	5w	BV/TV, mean density of BV	N	Nitially trapped in lungs for about 8–9 days and then gradually redistributed to the fracture site
Jiang et al., 2019 [55]	C57BL/6 mice	6w	N	Female	12	Stabilized femur fracture	BMSCs	N	1 × 10^5^	N	Tail vein	Once	At the same time of transplantation	2w	BMD, BV, Bone bass	Parathyroid hormone 1–34	Homing to bone callus
Myers et al., 2012 [58]	Mice	N	N	N	7	Fracture model	BMSCs	≤P5	1 × 10^6^	N	Tail vein	Once	After fracture	2w	New bone area	Insulin-like growth factor	Homing to the site of injury
Rapp et al., 2015 [56]	C57BL/6 mice	12w	N	Male	6	Fracture model	BMSCs	P4-P6	1 × 10^6^	N	Tail vein	Twice	After 2 h of fracture	21d	BV/TV	N	Recruiting into the evolving fracture callus and the transgene was also detected in the lung, heart, liver and kidneys
Tanriverdi et al., 2020 [53]	Wistar-albino rats	10–12w	300–350g	N	7	Polytrauma model	BMSCs	P3	3–3.5 × 10^5^	1 × 10^6^/kg	IV injection	Twice	After 36 h or 5 days of fracture	21d	areas for bone	N	N
Wang et al., 2018 [54]	C57BL/6 mice	8–10w	N	Male	5	Unilateral transverse femur fracture	BMSCs	P3-P4	1 × 10^6^	N	Tail vein	Once	After 1 or 7 or 14 days of facture	2w/6w	BV/TV, BMD	N	Arriving at a fracture site via the lung
Weaver et al., 2010 [50]	Sprague-dawley rats	6m	N	Male	13	Femur fractures	BMSCs	N	1 × 10^5^	N	Tail vein	Once	Before the first application of axial displacement	10/24/48d	BMD	N	Migrating to the femora
Wilson et al., 2012 [48]	Swine	6m	60–80kg	Male	5	Mandible defect	ADSCs	N	5 × 10^6^	6.25–8.3 × 10^4^/kg	Ear vein	Once	At the same time of transplantation	4w	BMD, BMC	N	Travelling to the site of injury
Yao et al., 2016 [52]	Mice	2m	N	Both	8–16	Closed transverse diaphyseal fracture	ADSCs	N	3 × 10^5^	N	Tail vein	Once	After 1 day of fracture	42d	BMD	LLP2A-Alendronate	Homing to the fracture gaps
Chen et al., 2017 [47]	SD rats	7w	180–220g	Female	10	Osteoporosis	BMSCs	P3	5–6.2 × 10^5^	2.8 × 10^6^/kg	Tail vein	Once	After 2 months of making osteoporosis models	2w	BMD	N	N
An et al., 2013 [46]	Balb-c nude mice	10w	N	Female	8	OVX-induced bone loss	UCB-MSCs	P6-8	4 × 10^5^	N	Tail vein	4 times	After 1, 2, 8, and 9 days of OVX surgery	4w/8w	BMD, BV/TV	N	N
Kiernan et al., 2016 [44]	C57BL/6 mice and BALB/c backcross	N	N	Female	8	Sca-1 knock out (type II osteoporosis)	BMSCs	P1	2 × 10^6^	N	Tail vein	Once	N	6m	BV/TV	N	Detected in the long bones and the majority of donor signal was found in the lungs, liver, and spleen
Kumar et al., 2010 [45]	C57BL/6 mice	6w	N	Female	10	OVX-induced bone loss	BMSCs	P4-8	2 × 10^6^	N	Tail vein	5 times	After 5 consecutive days of surgery	5w/10W/15w	BMD	BMP-2	Homing of the transplanted cells to bone marrow accompanied by reduction in the number of cells homing to other tissues including lung, liver, kidney and lymph node
Li et al., 2013 [57]	Swine	12m	30–40kg	Male	5	Bisphosphonate-Related Jaw Osteonecrosis	BMSCs	N	1 × 10^6^	2.5–3.3 × 10^4^/kg	Ear vein	Once	After 8 weeks of tooth extractions	12w	Mean area of osteoid	N	Homing to bone
Sui et al., 2016 [40]	C57BL/6 mice	12w	20–22g	Female	4	Glucocorticoid-induced osteoporosis	BMSCs	N	1 × 10^6^	4.5–5 × 10^7^/kg	IV injection	Once	After 7 days of GIOP injection	5W	BV/TV,BMD	N	Homing to recipient bone marrow within 24 h postinfusion and engrafted for at least 4 weeks postinfusion
Sui et al., 2018 [41]	C57BL/6 mice	12w	N	Female	5	OVX-induced bone loss	BMSCs	N	1 × 10^7^	N	IV injection	Once	After 4 weeks of surgery	4W	BV/TV	N	Less than 5% of the total gonadectomy marrow area in the first 24 h and declined to only approximately 2% at the end of the observation period
Wang et al., 2020 [42]	C57BL/6 mice and Balb/c mice	N	N	Female	7	OVX-induced bone loss	Uterine stem cell-derived osteoprogenitor cells	N	1 × 10^5^	N	Tail vein	8 times	Once a week up to a total of 8 weeks after ovariectomy surgery	6w	BMD, BV/TV,	N	Homing to the femoral head
Yao et al., 2013 [43]	C57BL/6 mice	2m	N	Female	8–14	OVX-induced bone loss	BMSCs	N	5 × 10^5^	N	IV injection	Once	After 2 weeks of OVX surgery 24-week old or 24-month old mice	6w	BV of LVB	Synthetic peptidomimetic ligand	Homing to bone marrow and bone surface

*BMSCs*, bone marrow-derived mesenchymal stem cells; *BV/TV*, bone volume (BV) to tissue volume (TV); *BMD*, bone mineral density; *hDPSCs*, human dental pulp stem cells; *IV*, intravenous; *ADSCs*, adipose-derived mesenchymal stem cells; *BMC*, bone mineral content; *UCB-MSCs*, umbilical cord blood, mesenchymal stem cells; *OVX*, ovariectomy; *LVB*, lumbar vertebral body

### Risk of bias

**Fig. 2:**
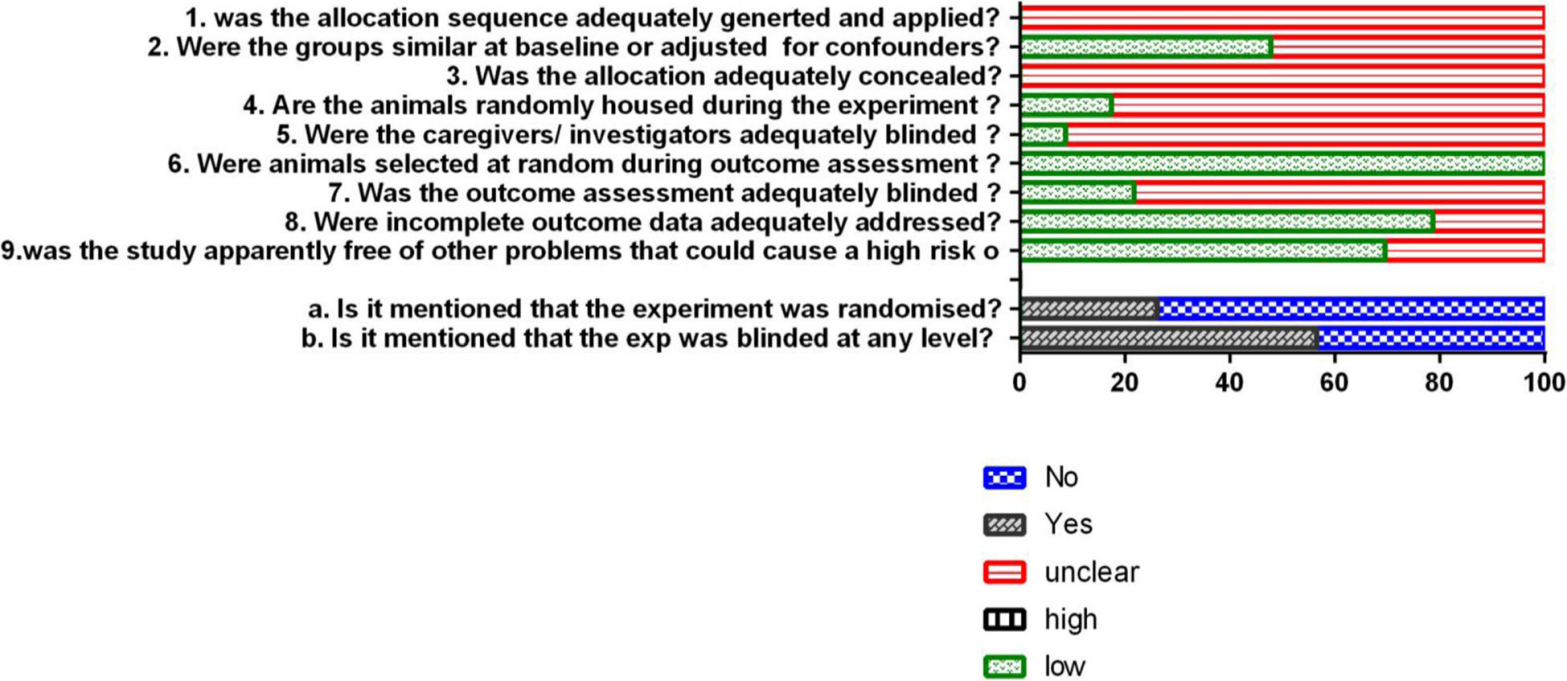
Risk of bias

Assessment of risk of bias for included studies was assessed and listed in Fig. 2. 56.5% of these studies reported randomization of the group designing. But none of these articles described the methods of randomization and the method used to conceal the allocation sequence, so the allocation sequence inadequately generated and applied. Almost half of the included studies described that all groups were in the same conditions at the start of experiments. And only 17.4% of studies randomly housed the animals during the experiment. Moreover, all studies were defined as low risk of bias of “random outcome assessment” for performance deviation items since the effect of experiment groups and control groups were evaluated at the same time. Meanwhile, 73.9% of experiments failed to mention that the experiment was blinded even 91.3% of studies without blinding of housing procedures and operators. In final analyzation of outcomes, 21.7% of studies reported outcome assessors were blinded to different groups and 21.8% of studies did not report the incomplete outcome data. In addition, the majority of studies scored at low risk in the bias of other problems. In conclusion, selection bias and performance bias were scored at unclear risk, but detection bias and attrition bias were almost scored at low risk.

### Meta-analysis

Twenty-one researches were included and three main outcomes were selected in this meta-analysis, including BMD (12 studies), BV/TV (10 studies), and percentage of new bone area (4 studies).

#### The main parameter: BMD

**Fig. 3:**
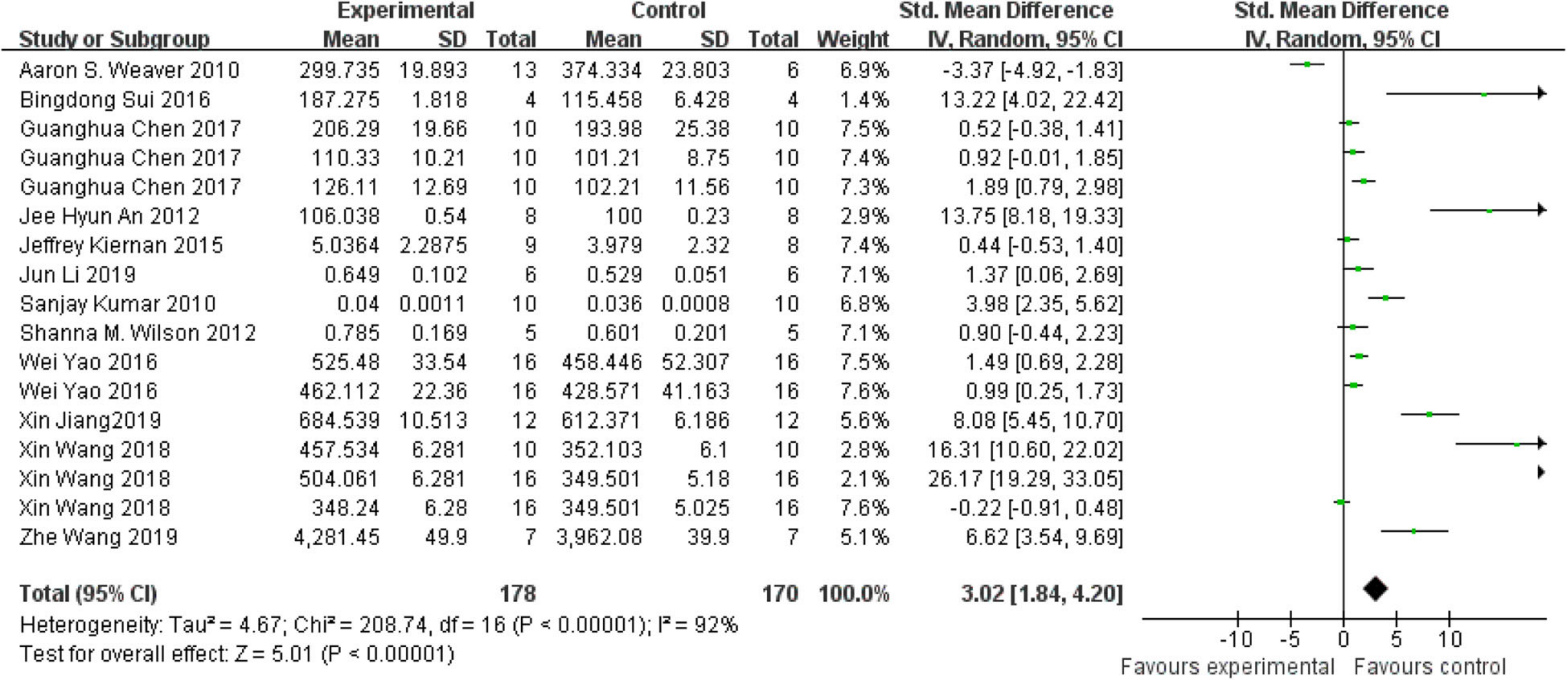
The forest plot: the effects of MSCs therapy on BMD, compared with controls. 95% CI, 95% confidence interval

BMD has always been an important index in evaluating bone regeneration [59]. The twelve identified references involved 348 animals (178 treated groups and 170 control groups) to evaluate BMD of systemic treatment of MSCs on new bone regeneration. Five experiments showed insignificant differences. Only one experiment displayed a negative effect on treated group and eleven experiments represented a positive effect on treated group compared with the corresponding control groups. Overall, there was a statistically significant beneficial effect of systemic treatment on new bone regeneration, as shown by the global estimate SMD and its 95% CIs (3.02 [1.84, 4.20]). But heterogeneity testing showed that I^2^=92%, indicating high heterogeneity (Fig. 3).

Subgroup analysis was carried out according to different animal species and gender, cell types, frequency and time of injection, and bone diseases. All analysis showed positive effect on systemic treatment except rat subgroup. And the analysis showed that the species of animal (p<0.0001) is the significant predictor of enhancing BMD, although the heterogeneity among studies was significant high (Table 2).

**Table 2:** BMD: stratified analysis of MSC-treated vs. control

	Subgroup	N	Effect estimate	I^2^	p*	p**
Species of animals	Mice	11	5.67 (3.78, 7.56)	94%	p<0.00001	p<0.0001
	Rat	5	0.34 (− 1.08, 1.76)	88%	p<0.00002	
	Swine	Not calculated				
Types of MSCs	BMSCs	12	3.23 (1.63, 4.84)	94%	p<0.00001	P=0.76
	Other MSCs	5	2.86 (1.13, 4.60)	87%	p<0.00001	
Frequency of injection	Single injection	14	2.20 (1.03, 3.37)	92%	p<0.00001	N
	Multiple injection	Not calculated				
Time of injection	Before modeling	Not calculated				N
	At the same time of modeling	Not calculated				
	After modeling	14	3.92 (2.59, 5.52)	93%	p<0.00001	
Bone diseases	Bone defect	12	2.26 (0.99, 3.54)	92%	p<0.00001	p=0.05
	Systematic bone diseases	5	6.25 (2.45, 10.05)	92%	p<0.00001	
Sex of animals	Male	6	3.64 (1.02, 6.27)	96%	p<0.00001	p=0.65
	Female	5	2.88 (0.90, 4.86)	89%	p<0.00001	

p* value for heterogeneity within each subgroup. p** value for heterogeneity between subgroups with meta-regression analysis. *MSCs*, mesenchymal stem cells; BMSCs, bone marrow-derived mesenchymal stem cells

#### BV/TV

**Fig. 4:**
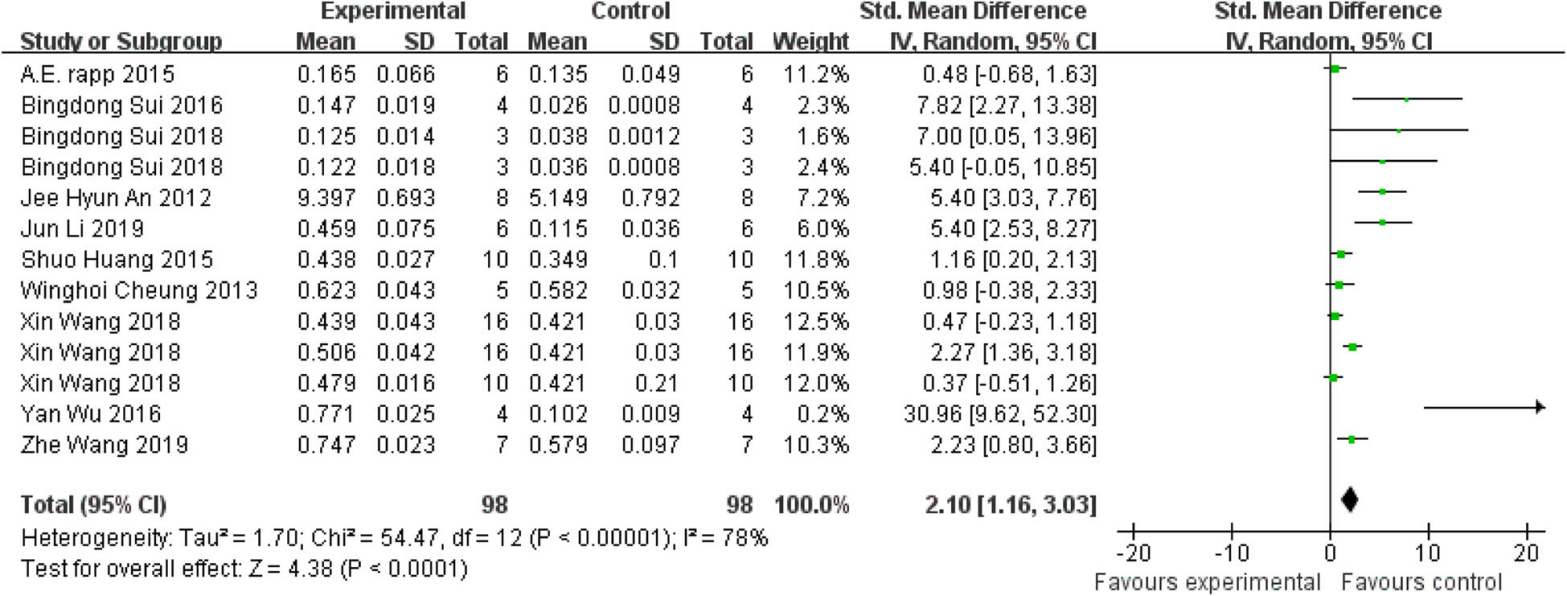
The forest plot: the effects of MSCs therapy on BV/TV, compared with controls. 95% CI, 95% confidence interval

The nine identified references involved 196 animals (98 treated groups and 98 control groups) to evaluate BV/TV. Five experiments presented insignificant differences, while eight experiments showed positive effect on treated group compared with control group. Overall, there was a statistically significant beneficial effect of systemic treatment on BV/TV, as shown by the global estimate SMD and its 95% CIs (2.10 [1.16, 3.03]). But heterogeneity testing still showed that I^2^=78%, indicating high heterogeneity (Fig. 4).

Subgroup analysis showed a beneficial effect of all subgroups. The subgroups of rat, other types of MSCs, multiple injections, injection while modeling, and female were not performed because the analysis required at least four independent comparisons. In addition, the analysis showed that bone diseases (p=0.03) are the significant predictor of enhancing BV/TV, although the heterogeneity among studies was significantly high (Table 3).

**Table 3:** BV/TV: stratified analysis of MSC-treated vs. control

	Subgroup	N	Effect estimate	I^2^	p*	p**
Species of animals	Mice	11	1.97 (0.98, 2.95)	78%	p<0.00001	N
	Rat	Not calculated				
Types of MSCs	BMSCs	10	1.55 (0.71, 2.39)	71%	p=0.0002	N
	other MSCs	Not calculated				
Frequency of injection	Single injection	10	1.96 (0.88, 3.03)	77%	p<0.00001	N
	Multiple injection	Not calculated				
Time of injection	At the same time of modeling	Not calculated				N
	After modeling	12	1.83 (0.92, 2.73)	76%	p<0.00001	
Bone diseases	Bone defect	8	1.77 (0.64, 2.89)	82%	p<0.00001	p=0.03
	Systematic bone diseases	5	4.27 (2.26, 6.27)	50%	p=0.09	
Sex of animals	Male	6	1 (0.12, 1.88)	75%	p=0.001	N
	Female	Not calculated				

p* value for heterogeneity within each subgroup. p** value for heterogeneity between subgroups with meta-regression analysis. *MSCs*, mesenchymal stem cells; *BMSCs*, bone marrow-derived mesenchymal stem cells

#### New bone formation

**Fig. 5:**
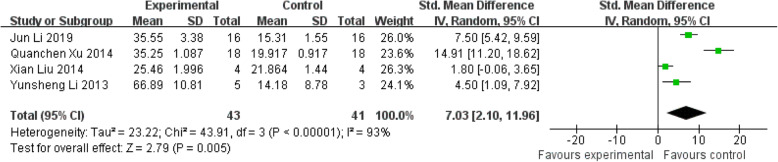
The forest plot: the effects of MSCs therapy on the percentage of new bone area, compared with controls. 95% CI, 95% confidence interval

The three identified references involved 84 animals (43 treated groups and 41 control groups) to evaluate the percentage of new bone area. Only one experiment found a statistically insignificant effect on new bone formation. Overall analysis showed that systemic strategies enhanced new bone formation, as shown by the global estimate SMD and its 95% CIs (7.03 [2.10, 11.96]). But heterogeneity testing showed that I^2^=93%, indicating the high heterogeneity (Fig. 5).

## Discussion

Twenty-three published animal researches with systemic treatment of MSCs were analyzed to investigate the effect of bone regeneration. The following is a brief summary of these results: (1) systemic application of MSCs promotes bone regeneration which was measured with BMD, BV/TV, or the percentage of new bone area. (2) Bone loss caused by systemic disease such as osteoporosis and osteonecrosis tended to produce a better response to systemic treatment. Although the difference was not statistically significant (p=0.05) in BMD, we believe that a statistically significant difference would be observed with more studies.

The meta-analysis results strengthened the evidence supporting systemic application of MSCs in bone regeneration. Only one study compared the efficacy of local and systemic treatments: Huang et al. [51] used femoral fracture models of mice to demonstrate that systemic and local application of MSCs promoted fracture healing equally by direct differentiate into osteoblasts. As we mentioned before, systemic bone disease has a better effect when using systemic injection. And local application is more suitable for bone fractures and bone defects.

Nowadays, the process of IV of MSCs in vivo was studied. MSCs are most initially trapped in lung microvasculature and rapidly phagocytosed by lung resident tissue macrophages. Then, MSCs may be recirculated and home to different organs, mostly liver due to the high circulating blood volume. At last, a small subset may redistribute to sites of injury or damage [60, 61]. However, intra-ventricular, which is a more invasive method, could deliver more MSCs to target sites. Besides, other methods of systemic administration include intra-arterial injection and intra-peritoneal administration but they were mostly used in stroke and cancer respectively [62]. In this systematic review, most of the included studies showed that MSCs could home to the site of bone defects or bone marrow, although some of them confirmed that most of the cells trapped in lungs, liver, kidneys, lymph nodes, and spleen (Table 1).

To date, the mechanism of stem cell transplantation therapy is based on the following: (1) MSCs still have the ability of homing to the injury site and differentiation into osteoblasts and chondrocytes directly although the number of these MSCs is limited [63–65]. (2) The paracrine effects of MSCs was secreting related factors such as cytokines, bone growth factors, chemokines to simulate angiogenesis and osteogenesis and recruiting host cells to the target sites [66, 67]. (3) It has been proved that MSCs have highly immunosuppressive functions which do not depend on the direct contact with immune cells, but on the recognition of MSCs by monocytic cells and evoking phagocytosis of macrophages [68]. In addition, MSCs can modulate the inflammatory micro-environment at the defect area and decrease the levels of interleukin (IL)-1β, IL-6, and tumor necrosis factor-α (TNF-α) [69]. Except non-specific immune suppressive effect, MSCs also has an effect on the specific immune system. On the one hand, MSCs can inhibit a series of activities of T cells, including the survival, activation, differentiation of T cell subsets, and even transformation of functional regulatory T (Treg) cells [70]. On the other hand, MSCs can also suppress several key steps of B cell-mediated immune responses, such as activation, proliferation, differentiation, and chemotactic responses [71]. As for systemic administration, many researchers who study the diseases of bone defect and bone fracture often only focus on the effect of MSCs for bone formation without explaining the specific mechanism [37, 48, 49]. Some of them verified MSCs could be recruited and home to defect sites to enhance new bone formation. Wu, Li, and Wang emphasized a key role of stromal cell-derived Factor-1/chemokine receptor 4 interaction in the migration of MSCs to the defect region [36, 38, 54]. Furthermore, more researches focus on the indirect effect of MSCs. The study by Yao et al. [52] reported that MSCs could alter the tissue microenvironment via paracrine signaling, secretion of chemokines, as well as angiogenic and anti-inflammatory factors. Wang et al. also concluded that MSCs could promote osteogenesis and bone calcification through the secretion of bone growth factors such as bone morphogenetic protein 2 and transforming growth factor-β1 [54]. Besides, Kumar et al. found that the paracrine effects of MSCs were essential to promote osteogenesis by inducing the expression of growth factors and cytokines in the local micro-environment and recruiting endogenous progenitor cells [45]. For immunosuppressive function, Li et al. reported that systemic MSC transplantation could suppress IL-17 and γδT cells and increase the level of Tregs in peripheral blood [57]. Sui et al. also inferred that systemically infused MSCs reduced total T-cell population and suppressed inflammation [41]. But these studies did not render unifying conclusions, so the mechanisms of systemic transplantation remain to be further elucidated.

In addition, some of the included studies mentioned combination treatment in this systematic review. Nowadays, a lot of researches have studied the combination treatment with MSCs in local application for bone regeneration. Compared with the local application of MSCs alone, the combined application of MSCs with protein molecules and scaffolds is usually more conducive to promoting bone regeneration [72, 73]. However, combination treatment in systemic applying of MSCs for bone engineering is a less explored area of research. Most of researches focus on protein molecule or ultrasound which could promote the effects on the angiogenesis efficiency of MSCs, such as erythropoietin (EPO) [38], LIPUS [49], and PTH1-34 [55]; some combination treatments could have a direct effect on osteogenesis, such as IGF-I [58] and BMP2 [45]; some protein molecules could increase the homing of transplanted MSCs to bone, such as LLP2A-Ale [43, 52] and PTH1-34 [55]. Besides, hypoxic was detected to be an effective way to improve the survival rate, recruitment, and osteogenesis of MSCs in transplantation, and its effects were mainly achieved through the SDF-1/CXCR4 axis [36]. Further researches are needed to evaluate the combination therapy of MSCs in order to discover more effective potential bone regeneration treatments.

However, we acknowledge some limitations regarding this systematic review. First, the assessment of risk of bias revealed that the body of the evidence was generally at a low quality, because RoB was scored “unclear” in the selected studies. As shown in Fig. 2, few articles reported the most important methods to avoid bias, such as randomization and blinding. For example, neither the methods of randomization nor whether the outcome assessment was blinded were reported in these included studies. This will increase the substantial risk of misunderstanding the effect of systemic therapy of MSCs on bone regeneration. Second, the included researches were differed from animal species and sex, bone disease model, the number and types of cells, the healing time, and so on. Therefore, high statistical heterogeneity was found in this meta-analysis. Random effects model was used to account for these results, and subgroup analyses (animal species, sex, bone disease and cell types) were performed in these analyses, but failed to reduce the heterogeneity. So, more comprehensive studies are needed to further evaluate these results. Third, due to the low quality of evidence, the results of this systematic review should be interpreted with caution. In the process of this systematic review, we have tried to reduce bias in the following ways: independent screening, data extraction, independent evaluation of results, and risk of bias evaluation.

Nowadays, few complete clinical trials for bone regeneration with systemic treatment has been reported until now while two studies were submitted in the web of clinical trials. Both of them focus on osteoporosis patients while allogenic MSC form umbilical cord and fucosylated BMSCs were used respectively in these studies. However, there are some problems still need to be discussed before adapting the systemic treatment of bone regeneration to clinical application. First of all, the type of MSCs used is one of the most important issues. BMSCs are the most commonly used in this systematic review, and they are also widely used for bone regeneration purposes. Because of the limited number of studies using other types of MSCs, we designed the studies grouped by BMSCs and other types of MSCs. And under the subgroup analysis, BMSCs displayed a favorable effect on bone regeneration, although there is no significant difference between the two subgroups. But this result should be taken with caution because of the high heterogeneity and small amounts of studies. Second, the passage number of MSCs used in treatment requires an objective measurement standard. MSCs ranging from passage 1 to passage 8 were conducted in this systematic review. Because the low frequency of MSCs we can acquired from the donor tissue, in vitro expansion is necessary for clinical use [74]. MSCs with a high number of passages enter senescence and begin to lose their stem cell characteristics such as proliferation and differentiation capabilities [75, 76]. As a result, the passage number is crucial to the final outcomes, whereas nearly half of the included studies ignore this important information. Thus it is strongly recommended that the passage number of MSCs should be clarified in future researches. Besides, the optimal cell dose is still unclear. MSCs with a concentration ranging from 6.25 × 10^4^/kg to 4 × 10^8^/kg were used in this systematic review; none of them studies the effect of different concentrations on bone regeneration. In this review, 15 included studies neglected to record body weight and only recorded the total number of transplanted cells. For the moment, cell dosing in clinical studies is based on the weight of patients, generally 1 × 10^5^/kg, not more than 1.2 × 10^6^/kg [61]. Therefore, it is necessary to display the weight of animals to provide references for clinical application. In addition, the cell dosing of different animals varied greatly. Both Li and Wilson et al. [48, 57] used swine models and the cell dosing are 2.5–3.3×10^4^/kg and 6.25–8.3×10^4^/kg respectively, while others who chosen rodents as experimental subjects are ranging from 1 × 10^5^/kg to 2 × 10^7^/kg. As a result, further studies should discuss the optimal dosing of MSCs in different species because the improper dosing may cause different responses. Moreover, most animal models selected in these systematic reviews are rodents (20 of 23 studies). This result may be due to the fact that rodents are easy to get and house, which tend to be selected to preliminary screenings. However, they have many anatomical and physiological differences with humans. And large animals, such as swine, should be used for validation of new therapy in the last phase, since large animals have more similarities with humans [77].

The time and frequency of injections are also issues that we need to pay attention to. Most of the existing studies use a single injection, but we found that multiple injections may have better outcomes in the evaluation of BMD, though meta-analysis has not been performed due to too few studies. Therefore, comparing the effects of single injection and multiple injections on bone regeneration is a future research direction. As for the injection time point, when we observe the preventive effect of bone loss, injection was normally conducted before or at the same time as the operation, while injection after surgery showed a therapeutic effect. However, most studies focus on the therapeutic role of MSCs in bone regeneration. Therefore, future researches may also be extended to the systemic therapy effect of MSCs to prevent bone loss, which is a promising treatment strategy to prevent osteoporosis caused by hormone changes or aging.

In summary, before clinical application, more researches on the systemic application of MSCs for bone regeneration should be carried out. Not only should we pay attention to application protocol of MSCs, but also explore how to transform the models into large animals.

## Conclusion

Based on the data of this meta-analysis, systemic application of MSCs promote bone regeneration compared with control groups, by assessing the treatment outcomes including BMD, BV/TV, or the percentage of new bone area. The role of MSCs in bone formation may include homing and differentiation, angiogenesis, inflammation, and immune response. However, due to the limitations inherent in the design of most included researches in this systematic review, there is still a long way to go before systemic treatment of MSCs for bone regeneration can be applied to the clinic. The results of this systematic review provide some certain reference for future experiments.

## Supplementary Information

**Additional file 1.** (DOCX 18 kb)

## Data Availability

All supporting data are included in the article and its additional files.

## Abbreviations

MSCs: Mesenchymal stem cells
SMD: Standard mean difference
BMSCs: Bone marrow-derived mesenchymal stem cells
ADSCs: Adipose-derived mesenchymal stem cells
GMSCs: Gingiva-derived mesenchymal stem cells
DPSCs: Dental pulp stem cells
IV: Intravenous
BMD: Bone mineral density
CT: Computed tomography
SYRCLE: Systematic Review Centre for Laboratory animal Experimentation
RoB: Risk of bias
CI: Confidence intervals
OVX: Ovariectomy
UCB: Umbilical cord blood
IL: Interleukin
TNF-α: Tumor necrosis factor-α
Treg: Regulatory T
