# Supplementary material for: Systemic therapy of MSCs in bone regeneration: a systematic review and meta-analysis
Source: Stem Cell Res Ther. 2021 Jul 2;12:377. doi: 10.1186/s13287-021-02456-w (PMC8254211; doi:10.1186/s13287-021-02456-w)
Supplement: Supplementary file 1 — Additional file 1. (DOCX 18 kb) [file 13287_2021_2456_MOESM1_ESM.docx]

| pubmed |  |
| --- | --- |
| Component 1: bone regeneration | ((((((bone formation[Title/Abstract]) OR (bone defect[Title/Abstract])) OR ((((Bone Regenerations[Title/Abstract]) OR (Regeneration, Bone[Title/Abstract])) OR (Regenerations, Bone[Title/Abstract])) OR (Osteoconduction[Title/Abstract]) )) OR (bone regeneration[MeSH Terms])) OR (bone tissue engineering[Title/Abstract])) OR (((((((Broken Bones[Title/Abstract]) OR (Bone, Broken[Title/Abstract])) OR (Broken Bone[Title/Abstract])) OR (Bone Fractures[Title/Abstract])) OR (Bone Fracture[Title/Abstract])) OR (Fracture, Bone[Title/Abstract])))) OR ("Fractures, Bone"[Mesh]) |
| Component 2:  Mesenchymal stem cells | ((((((((((((((((((((((((((((((((((Stem Cell, Mesenchymal[Title/Abstract]) OR (Stem Cells, Mesenchymal[Title/Abstract])) OR (Mesenchymal Stem Cell[Title/Abstract])) OR (Bone Marrow Mesenchymal Stem Cells[Title/Abstract])) OR ( [Title/Abstract])) OR (Bone Marrow Stromal Cell[Title/Abstract])) OR (Bone Marrow Stromal Cells, Multipotent[Title/Abstract])) OR (Multipotent Bone Marrow Stromal Cells[Title/Abstract])) OR (Adipose-Derived Mesenchymal Stem Cells[Title/Abstract])) OR (Adipose Derived Mesenchymal Stem Cells[Title/Abstract])) OR (Mesenchymal Stem Cells, Adipose-Derived[Title/Abstract])) OR (Mesenchymal Stem Cells, Adipose Derived[Title/Abstract])) OR (Adipose-Derived Mesenchymal Stromal Cells[Title/Abstract])) OR (Adipose Derived Mesenchymal Stromal Cells[Title/Abstract])) OR (Adipose Tissue-Derived Mesenchymal Stem Cells[Title/Abstract])) OR (Adipose Tissue Derived Mesenchymal Stem Cells[Title/Abstract])) OR (Adipose Tissue-Derived Mesenchymal Stromal Cells[Title/Abstract])) OR (Adipose Tissue Derived Mesenchymal Stromal Cells[Title/Abstract])) OR (Mesenchymal Stromal Cells[Title/Abstract])) OR (Mesenchymal Stromal Cell[Title/Abstract])) OR (Stromal Cell, Mesenchymal[Title/Abstract])) OR (Stromal Cells, Mesenchymal[Title/Abstract])) OR (Multipotent Mesenchymal Stromal Cells[Title/Abstract])) OR (Mesenchymal Stromal Cells, Multipotent[Title/Abstract])) OR (Mesenchymal Progenitor Cell[Title/Abstract])) OR (Mesenchymal Progenitor Cells[Title/Abstract])) OR (Progenitor Cell, Mesenchymal[Title/Abstract])) OR (Progenitor Cells, Mesenchymal[Title/Abstract])) OR (Wharton Jelly Cells[Title/Abstract])) OR (Wharton's Jelly Cells[Title/Abstract])) OR (Wharton's Jelly Cell[Title/Abstract])) OR (Whartons Jelly Cells[Title/Abstract])) OR (Bone Marrow Stromal Stem Cells[Title/Abstract])) OR (bone marrow stem cells[Title/Abstract])) OR ("Mesenchymal Stem Cells"[Mesh])) OR (("Stem Cells"[Mesh]) OR (((((((((((((((Cell, Stem[Title/Abstract]) OR (Cells, Stem[Title/Abstract])) OR (Stem Cell[Title/Abstract])) OR (Progenitor Cells[Title/Abstract])) OR (Cell, Progenitor[Title/Abstract])) OR (Cells, Progenitor[Title/Abstract])) OR (Progenitor Cell[Title/Abstract])) OR (Mother Cells[Title/Abstract])) OR (Cell, Mother[Title/Abstract])) OR (Cells, Mother[Title/Abstract])) OR (Mother Cell[Title/Abstract])) OR (Colony-Forming Unit[Title/Abstract])) OR (Colony Forming Unit[Title/Abstract])) OR (Colony-Forming Units[Title/Abstract])) OR (Colony Forming Units[Title/Abstract]))) |
| Component3: systematically application | ((((home[Title/Abstract]) OR (homing[Title/Abstract])) OR (systematic*[Title/Abstract])) OR (vein[Title/Abstract])) OR (Transplant*[Title/Abstract]) |
| Component4:  Animal model | Animal filter |

2987

| embase |  |
| --- | --- |
| Component 1: bone regeneration | “bone formation”:ab,ti OR “bone defect”:ab,ti OR “Bone Regenerations”:ab,ti OR “Regeneration, Bone”:ab,ti OR “Regenerations, Bone”:ab,ti OR “Osteoconduction”:ab,ti OR “bone regeneration”/exp OR “bone tissue engineering”:ab,ti OR “Broken Bones”:ab,ti OR “Bone, Broken”:ab,ti OR “Broken Bone”:ab,ti OR “Bone Fractures”:ab,ti OR “Bone Fracture”:ab,ti OR “Fracture, Bone”:ab,ti OR "Fractures "/exp |
| Component 2:  Mesenchymal stem cells | “Stem Cell, Mesenchymal”:ab,ti OR “Stem Cells, Mesenchymal”:ab,ti OR “Mesenchymal Stem Cell”:ab,ti OR “Bone Marrow Mesenchymal Stem Cells”:ab,ti OR “Bone Marrow Mesenchymal Stem Cell”:ab,ti OR “Bone Marrow Stromal Cell”:ab,ti OR “Bone Marrow Stromal Cells, Multipotent”:ab,ti OR “Multipotent Bone Marrow Stromal Cells”:ab,ti OR “Adipose Derived Mesenchymal Stem Cells”:ab,ti OR “Mesenchymal Stem Cells, Adipose Derived”:ab,ti OR “Adipose Tissue Derived Mesenchymal Stem Cells”:ab,ti OR “Adipose Tissue Derived Mesenchymal Stromal Cells”:ab,ti OR “Mesenchymal Stromal Cells”:ab,ti OR “Mesenchymal Stromal Cell”:ab,ti OR “Stromal Cell, Mesenchymal”:ab,ti OR “Stromal Cells, Mesenchymal”:ab,ti OR “Multipotent Mesenchymal Stromal Cells”:ab,ti OR “Mesenchymal Stromal Cells, Multipotent”:ab,ti OR “Mesenchymal Progenitor Cell”:ab,ti OR “Mesenchymal Progenitor Cells”:ab,ti OR “Progenitor Cell, Mesenchymal”:ab,ti OR “Progenitor Cells, Mesenchymal”:ab,ti OR “Wharton Jelly Cells”:ab,ti OR “Whartons Jelly Cells”:ab,ti OR “Bone Marrow Stromal Stem Cells”:ab,ti OR “bone marrow stem cells”:ab,ti OR "Mesenchymal Stem Cells"/exp OR "Stem Cells"/exp OR “Cell, Stem”:ab,ti OR “Cells, Stem”:ab,ti OR “Stem Cell”:ab,ti OR “Progenitor Cells”:ab,ti OR “Cell, Progenitor”:ab,ti OR “Cells, Progenitor”:ab,ti OR “Progenitor Cell”:ab,ti OR “Mother Cells”:ab,ti OR “Cell, Mother”:ab,ti OR “Cells, Mother”:ab,ti OR “Mother Cell”:ab,ti OR “Colony-Forming Unit”:ab,ti OR “Colony Forming Unit”:ab,ti OR “Colony-Forming Units”:ab,ti OR “Colony Forming Units”:ab,ti |
| Component3: systematically application | home:ab,ti OR homing:ab,ti OR “systematic*”:ab,ti OR vein:ab,ti OR “Transplant*”:ab,ti |
| Component4:  Animal model | 'animal experiment'/exp OR 'animal model'/exp OR 'experimental animal'/exp |

1054

Find duplicates ---3597
